# Modern MR Imaging Technology in Rectal Cancer; There Is More Than Meets the Eye

**DOI:** 10.3389/fonc.2020.537532

**Published:** 2020-10-08

**Authors:** Hester E. Haak, Monique Maas, Stefano Trebeschi, Regina G. H. Beets-Tan

**Affiliations:** ^1^Department of Radiology, Netherlands Cancer Institute, Antoni van Leeuwenhoek, Amsterdam, Netherlands; ^2^Department of Surgery, Netherlands Cancer Institute, Antoni van Leeuwenhoek, Amsterdam, Netherlands; ^3^GROW School for Oncology and Developmental Biology, Maastricht University, Maastricht, Netherlands; ^4^Department of Regional Health Research, University of Southern Denmark, Odense, Denmark

**Keywords:** rectal cancer, MR imaging, organ preservation, functional imaging, artificial intelligence

## Abstract

MR imaging (MRI) is now part of the standard work up of patients with rectal cancer. Restaging MRI has been traditionally used to plan the surgical approach. Its role has recently increased and been adopted as a valuable tool to assist the clinical selection of clinical (near) complete responders for organ preserving treatment. Recently several studies have addressed new imaging biomarkers that combined with morphological provides a comprehensive picture of the tumor. Diffusion-weighted MRI (DWI) has entered the clinics and proven useful for response assessment after chemoradiotherapy. Other functional (quantitative) MRI technologies are on the horizon including artificial intelligence modeling. This narrative review provides an overview of recent advances in rectal cancer (re)staging by imaging with a specific focus on response prediction and evaluation of neoadjuvant treatment response. Furthermore, directions are given for future research.

## Introduction

In the past, rectal cancer surgery was associated with a local recurrence rate of up to 30% (1). Due to the introduction of total mesorectal excision (TME) in combination with neoadjuvant (chemo-) radiotherapy and optimal staging by MR imaging (MRI), this rate is now down to less than 3% (2). MRI plays a pivotal role in primary staging to stratify the patient to the right treatment according to the risk for local recurrence. Restaging MRI after neoadjuvant treatment can accurately show downsizing and downstaging of the tumor and plan the surgical approach. In approximately 15–20% of the patients neoadjuvant chemoradiation (CRT) leads to (near) complete response. Organ preserving approach such as a watch-and-wait (W&W) has shown to be a safe alternative for surgery provided that the patient is managed in expert centers, equipped with modern imaging and endoscopic technology and a dedicated multidisciplinary team. To be able to safely apply W&W, selection of patients with a (near) complete response is key. Functional MRI techniques capture changes in tumor perfusion and microstructure before morphological changes become apparent (3). A well-established functional MRI is diffusion-weighted MRI (DWI) which analyses the movement or “diffusion” of water molecules in which tissues with high cellularity (i.e., tumors and lymph nodes) have restricted diffusion (high signal), while normal tissue and fibrosis will lead to free diffusion (low signal). DWI is now part of the standard restaging MRI. In addition, other techniques such as perfusion MRI [dynamic contrast enhanced (DCE) MRI] and artificial intelligence modeling are being explored. This narrative review provides an overview of recent advances in rectal cancer imaging with a specific focus on response prediction and evaluation of neoadjuvant treatment response. Furthermore, directions are given for future research.

### Baseline Imaging

MR imaging and endorectal ultrasound (EUS) are the established modalities for rectal cancer imaging. MRI is the most accurate modality to assess the tumor extent, nodal involvement, and guide treatment planning. For the distinction between T1 and T2 tumors, MRI is not accurate, therefore EUS is used for this specific purpose (4). However, morphological imaging does not provide information on tumor biology. Martens et al. reviewed the available literature on different volumetric methods and showed that whole-volume measurements on pre-CRT imaging reaches the highest accuracy of 71–73% for prediction of response to chemoradiotherapy (CRT) (5). Studies which focused on both MR and DWI volumetry at baseline, MRI showed low to moderate performances for predicting the response to CRT as compared to volumetric changes at post-CRT imaging (AUC of 0.57–0.73% vs. 0.63–0.77) (6, 7). The largest evidence is for DWI. A low pre-CRT apparent diffusion coefficient (ADC) at baseline DWI has by several studies shown to be significantly related with pathological complete response (pCR) and good response (6, 8–11). A possible explanation is that tumors with a high ADC value have more areas of necrosis which makes them less sensitive for radio- and chemotherapy (12). Despite some initial promising results for ADC subsequent literature showed conflicting results and considerable variability in reported cut off ADC values (13). Hence ADC measurements has not gained a significant role in response evaluation of rectal cancer treatment. Intravoxel incoherent motion (IVIM) uses low-*b*-values of DW-images to extract the perfusion fraction hence providing information on the tumor microvasculature without administration of intravenous contrast. Several studies show that IVIM is promising in prediction of response (14, 15). Diffusion kurtosis uses very high *b*-values and reflects intratumoral heterogeneity. The first few studies have not shown superiority of kurtosis imaging above ADC parameters in predicting the treatment response (16, 17). Two studies did show some potential for kurtosis imaging to predict response (18, 19). However, due to the lack of standardization and lack of strong evidence both IVIM and kurtosis imaging are currently only explored in a research setting (20).

DCE-MRI is a method which measures the inflow of intravenously injected contrast agents into the tumor and leakage of contrast into the extracellular space on T1W-MRI and provides direct information of the tissue perfusion. DCE-MRI can be analyzed quantitatively (by measurement of the perfusion of a voxel-by-voxel basis) or by semiquantitative analyses (in which a signal intensity time curve is plotted to assess parameters such as time to peak enhancement or area under the curve). DCE-MRI can provide valuable information on tumor biology (aggressiveness and the degree of angiogenesis) and initial studies have shown promise in the prediction of response (19, 21–25). Several studies showed that patients who achieved a pCR had significantly higher perfusion parameters [Ktrans, Kep (volume of extracellular space), and Ve (constant of flow rate)] than those who did not (21, 22, 25). Another group showed that the “late slope” of the signal enhancement curve after administration of contrast on baseline DCE-MRI was able to differentiate between good and poor responders with an AUC of 0.90 (23), although this study used a macromolecular blood pool contrast agent “gadofosveset” instead of the in clinics routinely applied micromolecular contrast “Gadolinium DTPA.” So far, DCE-MRI has not found its way to clinical practice due to the relatively high intra- and inter-tumor variation, need for intravenous contrast agents and lack of robustness of the technique. Research for optimization as well as standardization of the technique is much needed (4, 26). Table 1 provides an overview of the accuracy and predictive values of the different MRI techniques in a primary setting before neoadjuvant treatment.

**TABLE 1:** Overview of the accuracy and predictive values of the different MRI techniques during baseline and restaging to identify pathological complete response.

	Sensitivity (%)	Specificity (%)	PPV (%)	NPV (%)	Accuracy (%)	AUC
**Baseline**						
T2W volumetry	31–55 (5–7)	74–83 (5–7)	31–50 (5–7)	79–88 (5–7)	71–73 (5–7)	0.57–0.73 (6,7)
DWI volumetry	57–65 (6,7)	76–78 (6,7)	37–50 (6,7)	82–91 (6,7)	72–74 (6,7)	0.63–0.77 (6,7)
ADC	38–69 (6,8,11,16)	68–86 (6,8,11,16)	35–42 (6,8,11,16)	78–91 (6,8,11,16)	66–81 (6,8,11,16)	0.55–0.77 (6,8,16,19)
IVIM						
Pseudo-diffusion coefficient Perfusion fraction	60 (14) 80 (14)	84 (14) 72 (14)	55 (14) 47 (14)	87 (14) 92 (14)	NA NA	0.74 (14) 0.71 (14)
Kurtosis						
Mean kurtosis coefficient Mean diffusion coefficient	93–100 (16,18,19) 64 (16)	67–81 (16,18,19) 62 (16)	62 (16) 36 (16)	97 (16) 84 (16)	84 (16) 63 (16)	0.86–0.91 (16,18,19) 0.59 (16)
DCE-MRI						
Kep Ktrans Ve	100 (25) 75–100 (21,25) 83 (19)	70 (25) 60–73 (21,25) 83 (19)	NA NA NA	NA NA NA	NA NA NA	0.67–0.75 (21,19,25) 0.57–0.92 (21,19,25) 0.66–0.86 (21,19)
Radiomics	80–96 (56,59)	67–89 (56,59)	NA	NA	93–95 (56)	0.66–0.97 (56–59,63)
**Restaging**						
T2W volumetry	14–100 (5–7, 30)	30–97 (5–7, 30)	26–82 (5–7, 30)	82–100 (5–7, 30)	44–86 (5–7, 30)	0.70–0.82 (6,7, 30)
DWI volumetry	70–79 (6,7,30)	95–100 (6,7, 30)	88–100 (6,7, 30)	80–94 (6,7, 30)	84–94 (6,7, 30)	0.91–0.93 (6,7, 30)
T2W-MRI fibrotic patterns	94 (31)	77 (31)	88 (31)	87 (31)	88 (31)	NA
ADC	46–93 (6,11,16, 30)	56–81 (6,11,16, 30)	27–71 (6,11,16, 30)	65–97 (6,11,16, 30)	53–78 (6,11,16, 30)	0.54–0.82 (6,7, 30)
IVIM						
True diffusion coefficient	60 (14)	97 (14)	86 (14)	89 (14)	NA	0.80 (14)
Kurtosis						
Mean kurtosis coefficient Mean diffusion coefficient	93 (16) 93 (16)	83 (16) 71 (16)	65 (16) 52 (16)	97 (16) 97 (16)	86 (16) 77 (16)	0.91 (16) 0.87 (16)
DCE-MRI						
Ktrans	83–100 (22)	67–78 (22)	29–33 (22)	97–100 (22)	NA	0.81–0.84 (22)
MT imaging	88 (41)	90 (41)	70 (41)	96 (41)	NA	0.96 (41)
3-modality approach	71 (42)	97 (42)	NA	NA	NA	0.89 (42)
Radiomics	100 (61)	91 (61)	72–90 (55,61)	95–100 (55, 61)	94 (55)	0.93–0.98 (55,61)

*The table provides an overview of studies who evaluated MR techniques during baseline or restaging to identify a pathological complete response. Studies who evaluated MR techniques to identify good responders vs. non-responders or parameters such as change values determined pre- and post-CRT were not selected. PPV = positive predictive value; NPV = negative predictive value; AUC = area under the curve receiver operating characteristics; T2W = T2-weigthed; DWI = diffusion-weighted MRI; ADC = apparent diffusion coefficient; IVIM = intravoxel incoherent motion; DCE-MRI = dynamic contrast enhanced MRI; Kep = rate constant; Ktrans = volume transfer constant; Ve = extravascular space; and MT imaging = magnetization transfer imaging.*

### Response Evaluation

As for primary staging, morphological MRI is also the main imaging modality to evaluate the luminal response and to identify extraluminal findings or remaining malignant nodes after neoadjuvant treatment. However, standard T2-weighted MRI lacks the ability to accurately evaluate response to neoadjuvant therapy because of the difficult distinction between fibrosis with and without viable tumor. Reported accuracies are 43–60% for detection of residual tumor after CRT (27–29). Studies addressing T2W and DWI volumetry have shown that the decrease in volume and absolute volume after CRT were correlated with response T2W volumetry and showed accuracies over 80% for the assessment of complete responders (6, 7). DWI volumetry using whole tumor volume manual delineation outperformed T2 volumetry (AUCs up to 0.93) in several studies (6, 7, 30). However, manual volumetry is time-consuming making it less useful to apply in clinical practice (13). Lambregts et al. proposed a method to qualitatively assess the fibrotic pattern that appears after CRT and showed that the exact type of fibrotic pattern on restaging T2W-MRI helps to evaluate the response after CRT (31). They found that the fibrotic pattern follows the pattern of the primary tumor. For example, a polypoid, or (semi)circular tumor shows a sharply demarcated semicircular fibrotic wall after CRT, and an irregular or spiculated tumor often shows irregular fibrotic thickening of the wall on restaging MRI. Only 25% of the patterns were easy to interpret, i.e., a normalized wall in the complete responders and bulky tumoral mass in the patients with residual disease. In the majority of the irradiated patients, however, different fibrotic patterns were seen (i.e., a mixed signal or irregular aspect on T2) which were difficult to interpret (31, 32). The magnetic resonance tumor regression grade (mrTRG), adapted from a similar TRG classification used in histopathology (33) categorized response into a scale from TRG-1 (only fibrosis, probably complete response) to TRG-5 (no fibrosis, probably residual disease) (34, 35). Siddiqui et al. showed that this metric has a good interobserver agreement and in 90% the radiologists correctly identified poor responders. However, in only 66% of the cases the radiologists correctly selected good responders (35). An on-going randomized controlled trial aims to assess the ability of mrTRG to direct management (36). The results of this trial will show whether mrTRG based stratification will impact outcome.

The value of functional MR parameters for response evaluation after CRT have been explored. Several studies showed that a higher value of ADC and a larger increase in ADC are both associated with a good response to CRT (10, 37, 38). A meta-analysis described pooled sensitivities and specificities of 68% and 69% for pretreatment ADC for the prediction of pCR after CRT, and of 72 and 78%, respectively, for the increase in ADC after CRT (11). One study showed that an increase of an IVIM coefficient was seen after CRT with a significantly higher value in good vs. poor responders (39). Another study showed that diffusion kurtosis imaging was feasible to assess response and superior to mrTRG (17). However, both techniques are far from ready to be implemented in clinics and remain in research setting (16, 17, 39).

A repeated finding in multiple DCE-MRI studies is that a large decrease of Ktrans after CRT is predictive for (complete) response (26). For most other (semi-) quantitative DCE parameters after CRT no robust conclusions can be drawn (26) which is the reason why DCE-MRI is not routinely applied in clinical practice. A less studied functional imaging technique is magnetization transfer (MT) imaging (traditionally applied in brain imaging). MT imaging explores differences in the magnetization interaction. The transfer of magnetization (MT ratio) between protons bound to macromolecules (collagen) and free/unbound water protons is high in case of collagen rich tissue (fibrosis) and may be useful to discriminate residual disease from post-CRT fibrosis (40, 41). Yet evidence is limited and MT imaging is only explored in research setting.

**FIGURE 1:**
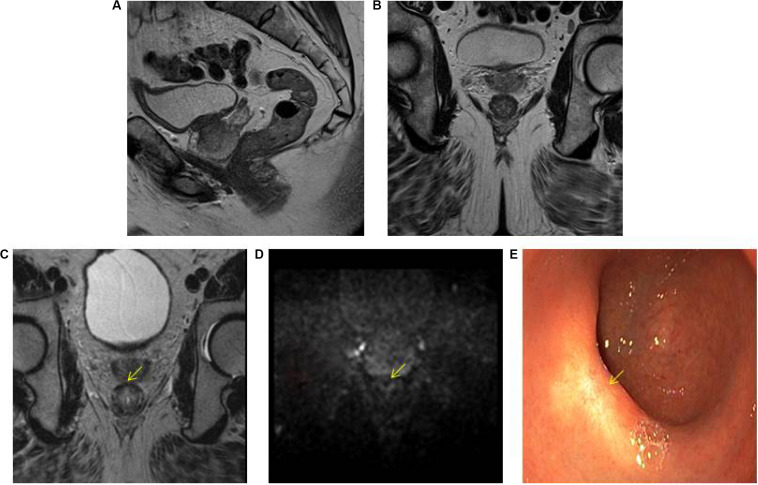
Three-modality approach with combination of T2W-MRI, DWI, and endoscopy of the selection of a patient with a clinical complete response. A low rectal tumor is seen on MRI **(A, B)**. On restaging MRI 8 weeks after completion of chemoradiotherapy **(C)** only minimal fibrosis (yellow arrow) is seen anteriorly in the rectal wall. On restaging DWI **(D)** there is absence of high diffusion signal (yellow arrow). Clinical assessment by endoscopy **(E)** reveals a white scar with telangiectasia (yellow arrow).

The highest accuracy to identify complete responders after CRT has been reached with the use of a three-modality approach (42) including digital rectal exam (DRE) with endoscopy, T2W-MRI, and DWI. Maas et al. described that when the combination of DRE, endoscopy, and T2W-MRI and DWI all indicate a complete response, this diagnosis is correct in 98% of the patients (42) Figure 1. Endoscopy was shown to be an invaluable tool for response evaluation, with MRI being an important adjunct to assess the extramural parts of the tumor and nodes. This method has been adopted globally in the selection of patients for organ preservation (43). Despite the good results, up to 15% of complete responders are still missed, due to the fact that many complete responding patients may show some findings that are often associated with residual tumor (e.g., ulcers at endoscopy, focal diffusion signal on DWI, irregular nodes on T2W-MRI among others) (44–46). Unfortunately, biopsies are of limited clinical value in this setting, because of the risk for sampling error and a risk for false positive findings (e.g., dysplasia in biopsy, but complete response in the TME specimen) (44, 46–48). An overview of the accuracy and predictive values of the different MRI techniques during restaging is given in Table 1.

### Artificial Intelligence

Artificial intelligence models hold promise in cancer imaging. These approaches aim to use computer algorithms to find associations between quantitative imaging features and clinical outcome. This procedure is termed *Radiomics*, and it can be carried out in a variety of different ways. The most common approach is to use pre-defined, general purpose quantitative imaging features that describe intensity distribution, tumor shape, and heterogeneity. More modern technique, such as deep learning, allows the computer to learn problem-specific imaging features leading to more robust models (49). Independently from the approach chosen, radiomics features can be combined with clinical and pathological data (possibly also extracted in the same fashion, i.e., pathomics) to predict clinical outcome, such as response to therapy (50). Across most radiomics studies, it is noted how non-visual information relating to tumor heterogeneity is an important biomarker for response prediction (51, 52). So far, radiomics has been applied to many tumor types (liver, lung, head and neck, and brain) using varying modalities (CT, MRI, 18F-FDG PET/CT) with promising results (50, 53, 54). Many studies have evaluated MR-based radiomics signatures (55–61), with a main focus on response evaluation. It is important to consider the technical challenges when applying radiomics on MR-images: problems with standardization, normalization and regularization of images may hamper the generalizability of radiomics models (62). Despite these difficulties, so far promising results have been found in response prediction (56–59, 63) and response evaluation (55, 60, 61, 64). Cui et al., for example, showed a favorable prognostic performance to predict pCR with radiomics on pre-CRT MR-images (AUCs of 0.94–0.97) (56), but other studies reported lower accuracies (AUCs of 0.69–0.79) (57–59, 63) (Table 1). Van Griethuysen et al. showed that radiomics on pre-CRT MR-images could predict response to therapy on image segmentation with comparable diagnostic performance as expert radiologists, regardless of their experience on image segmentation (63). On post-CRT MR T2W-images, Horvat et al. showed that radiomics had a better classification performance compared to the combination of DWI and T2W-MRI to identify pCR, with a significant higher specificity and PPV (91% vs. 56%; 72% vs 30%) (61). However, sensitivity and NPV were not significantly different. Another study concluded that radiomics could be used as an additional tool for clinical decision making on post-CRT imaging (64). Until now, only single center studies using a heterogeneous methodology have been performed. Additionally, external validation of findings in radiomics research is often lacking, which is an important prerequisite to eventually apply developed predictive radiomics models in clinical practice. Currently, initiatives are being taken to deal with standardization of radiomics analyses and start up large datasets in order to facilitate external validation by international collaborations.

## Discussion

During the past decades advances in MR imaging technology and in image analysis and post processing methods have opened new windows of opportunity for research that will foster further personalization of treatment. Patients with smaller tumors will undergo neoadjuvant treatment with the aim to achieve a complete response and to offer organ preserving treatment. Main clinical questions concern the ability of a non-invasive imaging tool to accurately select before the onset of treatment those patients who are likely to achieve a (near) complete response. It is expected that functional parametric MRI will perform superior to conventional MRI because the combination of morphological and functional data provides a comprehensive information on the tumor. More advanced metrics derived from DWI perfusion and kurtosis imaging as well as artificial intelligence modeling are promising but currently only the subject of research.

## Author Contributions

HH drafted the manuscript. ST helped to draft the artificial intelligence section. MM and RB-T helped to draft and review the manuscript. All authors read and approved the final manuscript.

## Conflict of Interest

The authors declare that the research was conducted in the absence of any commercial or financial relationships that could be construed as a potential conflict of interest.
